# The Importance of Fatty Acid Precision Nutrition: Effects of Dietary Fatty Acid Composition on Growth, Hepatic Metabolite, and Intestinal Microbiota in Marine Teleost *Trachinotus ovatus*

**DOI:** 10.1155/2023/2556799

**Published:** 2023-01-11

**Authors:** Guanrong Zhang, Lijun Ning, Kunsheng Jiang, Jun Zheng, Junfeng Guan, Hengji Li, Yongcai Ma, Kun Wu, Chao Xu, Dizhi Xie, Fang Chen, Shuqi Wang, Yuanyou Li

**Affiliations:** ^1^University Joint Laboratory of Guangdong Province, Hong Kong and Macao Region on MBCE, College of Marine Sciences, South China Agricultural University & Guangdong Laboratory for Lingnan Modern Agriculture, Guangzhou, 510642 Guangdong, China; ^2^Guangdong Provincial Key Laboratory of Marine Biotechnology, Shantou University, Shantou, Guangdong 515063, China

## Abstract

Our recent study demonstrated that diet with blend oil (named BO1) as lipid, which is designed on the base of essential fatty acid requirement of *Trachinotus ovatus*, achieved good performance. Here, to confirm its effect and investigate the mechanism, three isonitrogenous (45%) and isolipidic (13%) diets (D1-D3) only differing in dietary lipids, which were, respectively, fish oil (FO), BO1, and blend oil 2 (BO2) consisting of FO and soybean oil at 2 : 3, were formulated and used to feed the *T. ovatus* juveniles (average initial weight: 7.65 g) for 9 weeks. The results showed that the weight gain rate of fish fed D2 was higher than that of fish fed D3 (*P* < 0.05) and had no significant difference from that of fish fed D1 (*P* > 0.05). Correspondingly, compared with the D3 group, fish of the D2 group exhibited better oxidative stress parameters such as lower serum malondialdehyde content and inflammatory indexes in the liver such as the lower expression level of genes encoding four interleukin proteins and tumor necrosis factor *α*, as well as higher hepatic immune-related metabolites such as valine, gamma-aminobutyric acid, pyrrole-2-carboxylic acid, tyramine, l-targinine, p-synephrine, and butyric acid (*P* < 0.05). Furthermore, the intestinal probiotic (*Bacillus*) proportion was significantly higher, while the pathogenic bacteria (*Mycoplasma*) proportion was significantly lower in the D2 group than that in the D3 group (*P* < 0.05). The main differential fatty acids of diet D2 were close to those of D1, while the levels of linoleic acid and n-6 PUFA, as well as the ratio of DHA/EPA of D3, were higher than those of D1 and D2. These results indicated that the better performance of D2 such as enhancing growth, reducing oxidative stress, and improving immune responses and intestinal microbial communities in *T. ovatus* may be mainly due to the good fatty acid composition of BO1, which indicated the importance of fatty acid precision nutrition.

## 1. Introduction

Fish oil (FO) has a comprehensive fatty acid composition and is rich in n-3 long-chain polyunsaturated fatty acid (n-3 LC-PUFA) such as docosahexaenoic (DHA) and eicosapentaenoic (EPA) acids to meet the requirement of fish for essential fatty acids (EFA), which is a high-quality lipid source for fish compound feed [1]. However, FO are scarce and expensive, thereby limiting the sustainable development of the aquaculture industry [2, 3]. To relieve the pressure, producers often use the abundant and relatively low-priced terrestrial vegetable oils (VO) to replace FO, which easily resulted in disorder of lipid metabolism, serious fat accumulation, fatty liver, decrease in immunity and flesh quality, and even a mass death of fish in the current aquaculture industry [4, 5]. For example, high levels of olive oil and palm oil in diets could reduce the growth performance and antioxidant capacity and induce inflammation in large yellow croaker (*Larimichthys crocea*) [6, 7]. The reasons may be multiple for this. However, the unscientific fatty acid composition in feed caused by improper use of dietary lipids, resulting in the lack or insufficiency of EFA for cultured fish, may be the key or important reasons, we think.

In order to verify this view, in recent years, we have carried out a series of culture experiments in seawater cages with golden pompano *Trachinotus ovatus*, an important mariculture fish on the coast of south China. First, through research, we found out that its EFA requirement is LC-PUFA, and the proper dietary n-3 LC-PUFA level is 0.64%-2.10%, and the DHA/EPA ratio is about 1.4 [8–10]. Second, based on the EFA demand characteristics of *T. ovatus*, a blend oil (named BO1) was developed and used as the dietary lipid to make diet for a culture test, and the growth performance and health indexes showed that the effect of BO1 is even better than that of fish oil [11]. However, the mechanism for better performance of BO1 is still unknown.

Therefore, the present study tried to explore the mechanism by culture experiments using three kinds of isonitrogenous (45%) and isolipidic (13%) diets (D1-D3) only differing in dietary lipids, which were FO, BO1, and blend oil 2 (BO2) consisting of FO and soybean oil at a ratio of 2 : 3, respectively. After the *T. ovatu*s juveniles were fed with D1-D3 for 9 weeks, the growth performance, serum oxidative stress index, expression level of inflammatory genes in the liver, hepatic metabolomics, and intestinal microbiota were analyzed so as to investigate the mechanism of dietary fatty acid composition affecting culture performance. The results can not only deepen our understanding of the importance of fatty acid precision nutrition, especially the demand for EFA, but also be helpful to promote the establishment and application of theory and technology of fish fatty acid precision nutrition.

## 2. Materials and Methods

### 2.1. Experimental Diets

**Figure 1:**
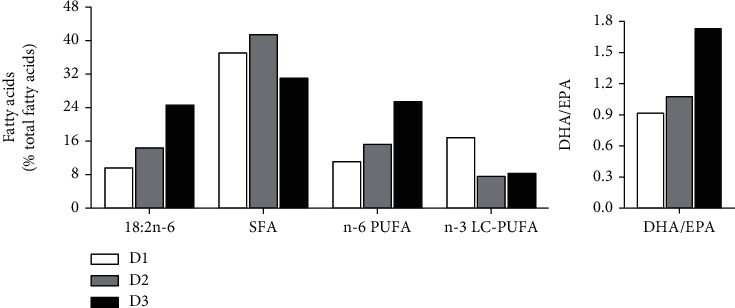
Main differential fatty acids of the experimental diets (% total fatty acids). SFA: saturated fatty acids; n-6 PUFA: n-6 polyunsaturated fatty acid; n-3 LC-PUFA: n-3 long-chain PUFA; EPA: eicosapentaenoic acid; DHA: docosahexaenoic acid.

Three isonitrogenous (45%) and isolipidic (13%) diets (D1-D3) differing only in lipid sources were formulated, which resulted in the difference in dietary fatty acid composition. The lipids of D1-D3 are FO, blend oil 1 (BO1) based on *T. ovatus*' demand characteristics for essential fatty acids [11], and blend oil 2 (BO2) consisting of FO and soybean oil at a ratio of 2 : 3 (a ratio commonly used in the production of *T. ovatus* feed in feed companies). Details of ingredients and proximate and fatty acid composition of the diets are presented in Tables 1 and 2 and Figure 1, respectively. All the dry ingredients were finely ground, well mixed, and homogenized. Then, the diets were produced as uniform pellets (2.5 mm) by an automatic pellet-making machine (SLC-45, Fishery Machinery and Instrument Research Institute, China) and air-dried to approximately 7% moisture. Finally, the diets were sealed in vacuum-packed bags and stored at -20°C until used.

### 2.2. Animals and Feeding

All animal care and use procedures in the present study were performed strictly in accordance with the standard operation procedures of the National Institutes of Health *Guide for the Care and Use of Laboratory Animals* (NIH Publications No. 8023, revised 1978) and approved by the Institutional Animal Care and Use Ethics Committee of South China Agricultural University.

*T. ovatu*s juveniles (approximate weight: 1.5 g) were purchased from a local fish farmer and cultivated with a commercial diet for seawater fish (crude lipid ≥ 5%, crude protein ≥ 40%, and ash ≤ 15%; Guangdong Feeds Group Co., Ltd, China) twice daily in floating sea cages (3 m × 3 m × 1.5 m) for four weeks at the coast near Nan Ao Marine Biology Station (NAMBS) of Shantou University. Before the experiment, the fish were acclimatized to the experimental conditions for 8 days and fed twice daily with a mixture of the three experimental diets in equal amounts. And then, a total of 270 healthy and almost the same size fish (average weight: 7.65 ± 0.06 g) were randomly distributed to 9 sea cages (1.0 m × 1.0 m × 1.5 m) with 30 fish per cage. Fish were fed to visual satiation twice daily (7:00 a.m. and 17:30 p.m.) for 9 weeks. During the feeding trial, water salinity and temperature fluctuated between 32‰ and 33‰, 25.0°C and 27.8°C, respectively. Besides, dissolved oxygen was about 6.2 mg L^−1^, and NH_4_^+^-N content was less than 0.1 mg L^−1^.

### 2.3. Sample Collection

At the end of the feeding trial, *T. ovatu*s were fasted for 6 h after the last feeding [12]; then, six fish per treatment were randomly selected from three replicate cages, anesthetized with 0.01% 2-phenoxyethanol and weighed, and then used to collect intestinal contents for intestinal microbiome analysis. The remaining fish was cultivated for two days and was fasted for 24 h after the last feeding, and then, their final weight was recorded to calculate final weight, weight gain rate (WGR), specific growth rate (SGR), and feed conversion ratio (FCR). Subsequently, twelve fish per treatment were randomly collected from three replicate cages and anesthetized for sampling. Among them, six fish were used for body proximate composition analysis, and another six fish were used for sampling of blood, liver, and muscle. Blood was collected from the caudal veins into 1.0 mL centrifuge tubes and stored at 4°C for 2 h. Then, serum was collected after the blood was centrifuged (3,500 g at 4°C for 10 min). Three liver samples about 0.5 g were collected from each fish for the analysis of hepatic metabolomics, fatty acid composition, and gene expression. One dorsal muscle sample was collected from each fish for the analysis of proximate composition. All the serum, liver, and muscle tissues were quickly frozen in liquid nitrogen and then stored at -80°C until analysis.

### 2.4. Analysis of Oxidative Stress-Related Parameters in Serum

The activity of catalase (CAT), superoxide dismutase (SOD), and glutathione peroxidase (GSH-Px), as well as the content of oxidized glutathione (GSSG) and malondialdehyde (MDA) in the serum, were measured according to the manufacturer instructions of the assay kits (Nanjing Jiancheng Bioengineering Co. Ltd, Nanjing, China).

### 2.5. Analysis of Proximate Composition and Fatty Acid Composition

Proximate composition (moisture, lipid, protein, and ash contents) of the diets and tissue samples were analyzed using the prescribed methods of Li et al. [13] and Ma et al. [14]. Fatty acid composition of the diets and tissue samples was determined following the methods detailed by Li et al. [8, 13]. Briefly, total lipid in each sample was extracted with chloroform/methanol (2 : 1 *v*/*v*) and methylated into fatty acid methyl esters (FAME) with KOH-methanol (0.5 M) and boron trifluoride methanol complex solution (15% BF3 basis, Sigma-Aldrich, USA). Then, the FAME were analyzed using a gas chromatograph (GC; Model 7890B, Agilent, USA) equipped with a hydrogen flame ionization detector and a capillary column [8, 13]. Finally, each fatty acid was identified by comparing the FAME profiles of the samples with known FAME standards (Supelco, 37 Component FAME mix C4-C24, Sigma No. CRM47885, Sigma-Aldrich, USA) and expressed as percentages of total fatty acids [8, 13].

### 2.6. Analysis of Real-Time Quantitative PCR

The expression levels of interleukin-1*β* (*il-1β*), interleukin-8 (*il-8*), interleukin-12 (*il-12*), interleukin-18 (*il-18*), and tumor necrosis factor *α* (*tnf-α*) genes in the liver of *T. ovatus* were determined by quantitative real-time polymerase chain reaction (qPCR), and the specific primers (Table 3) were designed according to the reference [15] and the genome sequences of *T. ovatus* (10.6084/m9.figshare.7570727.v3). The hepatic RNA extraction and reverse transcription were conducted using the prescribed methods of Zhang et al. [16]. The qPCR was carried out in a CFX Connect Real-Time System (Bio-Rad Laboratories, Inc., California, USA) in a total volume of 10 *μ*L containing 5 *μ*L SYBR® qPCR Mix (Toyobo Co., Ltd., Japan), 0.5 *μ*L each primer, 1 *μ*L cDNA, and 3 *μ*L ddH_2_O. The program consisted of a DNA denaturation of 95°C for 30 s, followed by 40 cycles at 95°C for 5 s and annealing at 60°C for 30 s, and then performed a melting curve of 0.5°C increments from 65 to 95°C. The relative expression of these genes was calculated by the 2^−*ΔΔ*Ct^ method [16].

### 2.7. Analysis of Hepatic Metabolomics

Six liver sample replicates of *T. ovatus* from each group were used for metabolomic analysis. The metabolite extraction and analysis were performed following the methods detailed by Duan et al. [17]. Briefly, the metabolites were detected using LC-MS (liquid-chromatography-mass spectrometry); mzXML format (xcms input file format) was obtained from the original data using Proteowizard software (v3.0.8789); the peak identification, peak filtration, and peak alignment were performed using R (v3.3.2) XCMS package (main parameter: bw = 2, ppm = 15, peakwidth = *c*(5, 30), mzwid = 0.015, mzdiff = 0.01, and method = centWave); base peak chromatograms were obtained according to the continuous description of the ions with the highest intensity in each mass spectrogram; then, all the data were determined using quality control and quality assurance [17]. After standardized treatment by autoscaling (mean-centering and scaling to unit variance, UV), a partial least squares-discriminant analysis (PLS-DA) model was used to determine the differential metabolites (DMs) between the pairwise comparison groups with a *P* value ≤ 0.05 combined with the first principal component of variable importance in projection (VIP) values (VIP ≥ 1), and all the metabolites were classified according to KEGG and Metabolon.inc [17]. DMs of D1 vs. D2, D1 vs. D3, and D2 vs. D3 were identified. Based on the exact mass match (error < 30 ppm) and secondary spectra MS/MS, metabolite identification was performed by searching the HumanMetabolome Database (HMDB) (http://www.hmdb.ca), massbank (http://www.massbank.jp/), Metlin (http://metlin.scripps.edu), LipidMaps (http://www.lipidmaps.org), mzclound (https://www.mzcloud.org), and company databases (Suzhou PANOMIX Biomedical Tech Co., LTD, China). Finally, the DMs of D2 vs. D3 were further identified according to VIP > 1.54 and *P* value < 0.05.

### 2.8. Analysis of Intestinal Microbiome

The bacterial DNA from the intestinal content of *T. ovatus* was firstly extracted by CTAB/SDS, the purity and concentration of DNA were detected by agarose gel electrophoresis, and the appropriate DNA was diluted with sterile water to 1 ng *μ*L^−1^. Then, use barcode-specific primers, 341F (5′-CCTAYGGGRBGCASCAG-3′) and 806R (5′-GGACTACNNGGGTATCTAAT-3′), and the diluted DNA template to amplify the V3-V4 region of 16S rDNA gene. All PCR were carried out using high efficiency and fidelity enzyme and Phusion® High-Fidelity PCR Master Mix with GC Buffer (New England Biolabs) to ensure amplification efficiency and accuracy. The PCR products were mixed equally and purified by the GeneJET Gel Extraction Kit (Thermo Scientific). The sequencing libraries were constructed using the TruSeq® DNA PCR-Free Sample Preparation Kit (Illumina, USA) and sequenced on the NovaSeq6000 platform.

The sequencing reads were assigned to each sample based on the unique barcode and truncated by cutting off the barcode and primer sequence, and then, the reads of each sample were merged with FLASH (Version 1.2.7, http://ccb.jhu.edu/software/FLASH/) to obtain the raw tags [12]. The raw tags were strictly filtered by QIIME (Version 1.9.1, http://qiime.org/scripts/split_libraries_fastq.html) to obtain high-quality clean tags. Then, the UCHIME algorithm is used to detect and remove chimeric sequences to obtain high-quality effective tags [18]. The UPARSE algorithm (Version 7.0.1001, http://www.drive5.com/uparse/) is used to cluster the effective tags of all samples into OTUs (operational taxonomic units) with 97% identity. Species annotation of OTU sequences was performed by the Mothur method and SSUrRNA Database of SILVA138 (http://www.arb-silva.de/) [18, 19]. Alpha diversity (observed-otus, Shannon, Simpson, Chao1, and Ace index) and beta diversity (principal component analysis, PCA) were calculated using QIIME (Version 1.9.1) and displayed with R (Version 2.15.3). Linear discriminant analysis (LDA) effect size (LEfSe) analysis was performed using LEfSe software to identify differential bacterial taxa within different groups [20], and the filter value of the LDA score was set at 4.

### 2.9. Correlation Analysis of Intestinal Bacteria and DMs

Pearson correlation analysis was conducted to reveal the correlation between intestinal bacteria and hepatic DMs of *T. ovatus* using the Cytoscape software coNet plug-in, and heat map was used to show the correlation between intestinal bacteria and hepatic metabolites; *P* < 0.05 indicated a statistically significant difference, *P* < 0.01 indicated very significant, and *P* < 0.001 indicated extremely significant [20].

### 2.10. Calculations and Statistical Analysis

The calculations of growth performance indexes such as the WGR, SGR, FCR, and survival rate (SUR) are as follows: WGR (%) = 100 × (final weight − initial weight)/(initial weight), SGR (%day^−1^) = 100 × [Ln(final weight) − Ln(initial weight)]/days, FCR = (total dry weight of feed fed)/(final weight − initial weight), and SUR (%) = 100 × survived fish number/total fish number.

All data were presented as the mean ± standard error of mean (SEM). One-way analysis of variance (ANOVA) was used to analyze the data with SPSS software (Version 20.0, USA). Duncan's multiple range test was used to analyze the significant differences among treatment means, and the significant level was set at 0.05.

## 3. Results

### 3.1. Growth Performance and Feed Utilization

As can be seen from Table 4, the final weight, SUR, and FCR of *T. ovatus* showed no significant differences among the three dietary groups (*P* > 0.05). Both the WGR and SGR of fish in the D2 group had no significant difference in the D1 group (*P* > 0.05), while they were significantly higher than those in the D3 group (*P* < 0.05).

### 3.2. Whole-Body and Muscle Proximate Composition

Data in Table 5 showed that the content of crude protein was higher, while that of crude lipid was lower in the whole-body and muscle of the D2 group than those of the D1 and D3 groups (*P* < 0.05). There is no significant difference between the D1 and D3 groups (*P* > 0.05). Also, the contents of ash in whole-body and muscle as well as the contents of moisture in muscle showed no significant difference among the three groups (*P* > 0.05).

### 3.3. Oxidative Stress-Related Parameters in Serum

**Figure 2:**
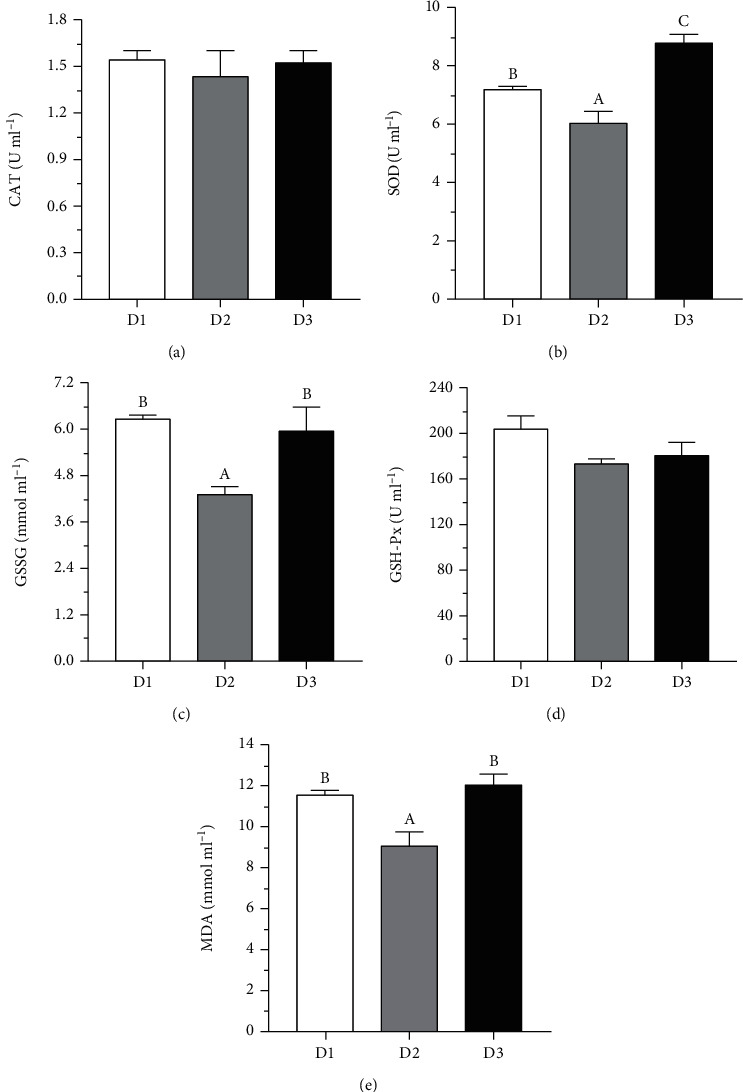
The serum parameters of oxidative stress in *T. ovatus* fed different diets. CAT: catalase; SOD: superoxide dismutase; GSH-Px: glutathione peroxidase; GSSG: oxidized glutathione; MDA: malondialdehyde. Data are means ± SEM from three treatments (*n* = 3). Each parameter bar not sharing a common letter indicates a significant difference (*P* < 0.05).

As showed in Figure 2, the serum SOD activity and GSSG and MDA contents in the D2 group were significantly lower than those in the D1 and D3 groups (*P* < 0.05). However, serum CAT and GSH-Px activities had no significant difference among the three groups (*P* > 0.05).

### 3.4. Immune Indices in Liver

**Figure 3:**
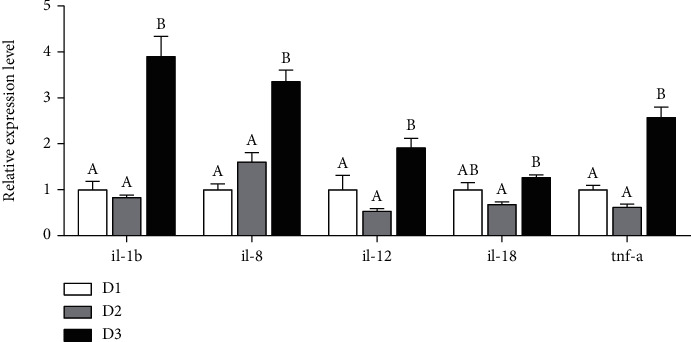
The expression levels of genes involved in innate immunity in the liver of *T. ovatus* fed different diets. *il-1β*: interleukin-1*β*; *il-8*: interleukin-8; *il-12*: interleukin-12; *il-18*: interleukin-18; *tnf-α*: tumor necrosis factor *α*. Data are means ± SEM from three treatments (*n* = 3). Each parameter bar not sharing a common letter indicates a significant difference (*P* < 0.05).

As showed in Figure 3, the expression levels of hepatic *il-1β*, *il-8*, *il-12*, *il-18*, and *tnf-α* genes in the D2 group were significantly lower than those in the D3 group (*P* < 0.05), but there was no significant difference between the D1 and D2 groups (*P* > 0.05).

### 3.5. Fatty Acid Composition of Diets and Liver

As can be seen from Figure 1, the main differential fatty acids between diets D2 and D1 were similar, whereas the proportion of linoleic acid (LA) and n-6 PUFA as well as the ratio of DHA/EPA of D3 were obviously higher, but its saturated fatty acid (SFA) proportion was lower than those of D1 and D2.  Table 6 shows that the hepatic 16:1, arachidonic acids (ARA), EPA, DHA, n-3 PUFA, and n-3 LC-PUFA proportion of fish in the D2 group were significantly lower than those in the D1 group (*P* < 0.05), while the hepatic 18:1, LA, 20:2n-6, linolenic acid (ALA), 20:3n-3, and n-6 PUFA proportions and DHA/EPA ratio were significantly higher than that in the D1 group (*P* < 0.05); no significant difference was observed in hepatic 16:0, 18:00, SFA, and monounsaturated fatty acid (MUFA) proportions between the D1 group and the D2 group (*P* > 0.05). Moreover, lower LA, 20:2n-6, and n-6 PUFA proportion as well as DHA/EPA ratio and higher 18:1 and MUFA proportion were found in the D2 group than those in the D3 group (*P* < 0.05); other fatty acids showed no significant difference between the D2 and D3 groups (*P* > 0.05).

### 3.6. Hepatic Metabolome Analysis

**Figure 4:**
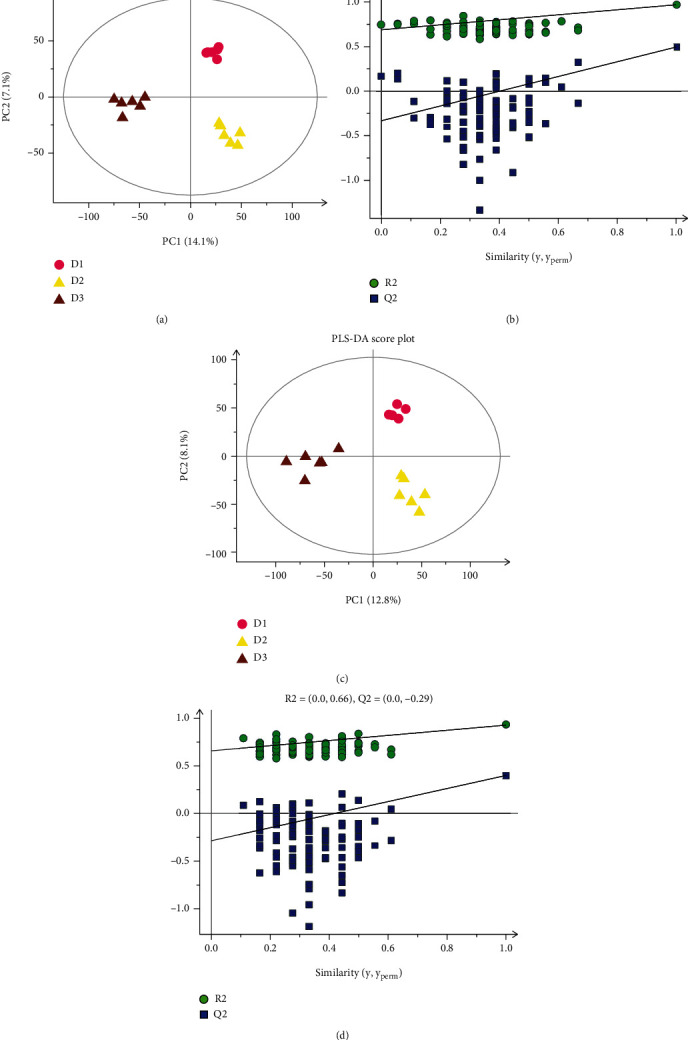
Derived PLS-DA score plots and corresponding permutation testing of PLS-DA from the LC-MS metabolite profiles in the liver of *T. ovatus* fed different diets: (a) PLS-DA score plot of positive ions; (b) permutation testing of positive ions; (c) PLS-DA score plot of negative ions; (d) permutation testing of negative ions.

**Figure 5:**
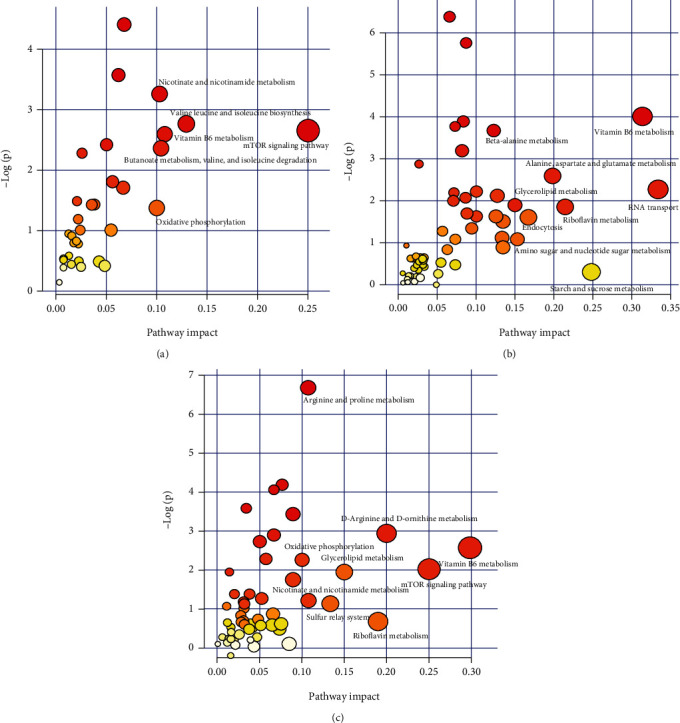
The most enriched KEGG pathway analysis of the DMs in the liver of *T. ovatus* fed the experimental diets D1, D2, and D3. The enriched pathway (a) of D1 vs. D2. The enriched pathway (b) of D1 vs. D3. The enriched pathway (c) of D2 vs. D3.

The alterations in hepatic metabolic profiles of *T. ovatus* fed different diets were explored according to metabolomic analysis. The PLS-DA score plot and permutation test displayed a significant difference among the three groups in both positive and negative ionization modes (Figure 4), suggesting that the lipid source caused hepatic metabolic phenotype alterations in *T. ovatus*. Then, to explore the potential metabolic pathways affected by lipid source, all DMs (VIP > 1, *P* < 0.05) were analyzed by KEGG annotation. Compared with the D1 group, the most enriched pathways of the D2 group were “mTOR signaling pathway,” “valine, leucine, and isoleucine metabolism,” and “vitamin B6 metabolism” (Figure 5(a)); those of the D3 group were “RNA transport,” “vitamin B6 metabolism,” and “alanine, aspartate, and glutamate metabolism” (Figure 5(b)). Compared with the D3 group, the most enriched pathways of the D2 group were “vitamin B6 metabolism,” “mTOR signaling pathway,” and “D-arginine and D-ornithine metabolism” (Figure 5(c)). A total of 61 DMs of D2 vs. D3 (VIP > 1.54, *P* < 0.05) were further identified in the liver using MS/MS analysis, and the largest category was “amino acids” (31.15%), followed by “carbohydrate” (14.75%) and “lipid” (14.75%) (Table 7), where some immune-related DMs, including pyrrole-2-carboxylic acid, gamma-aminobutyric acid (GABA), p-synephrine, butyric acid, l-valine, tyramine, and l-targinine, in the D2 group were higher than those in the D3 group.

### 3.7. Intestinal Microbiota Changes

**Figure 6:**
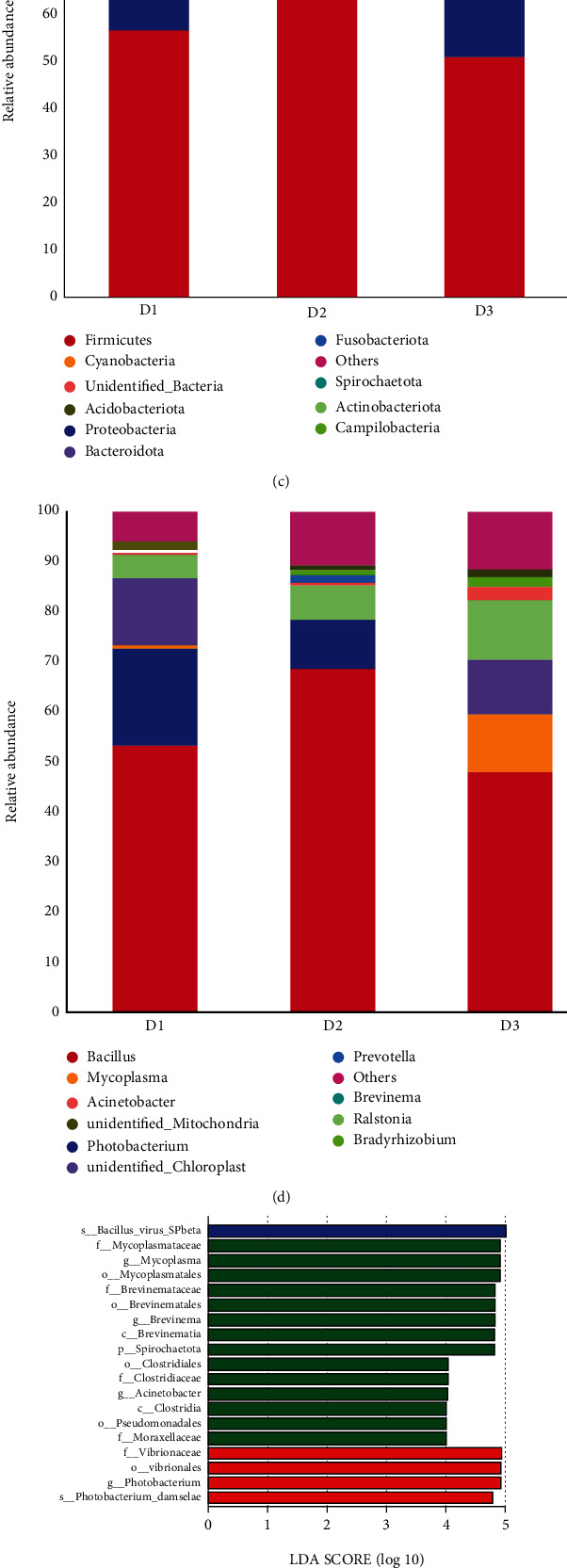
Comparison of intestinal microbial diversity and composition among *T. ovatus* fed the experimental diets D1, D2, and D3. PCoA plots based on weighted UniFrac metrics (a) and unweighted UniFrac metrics (b). Average relative abundances of dominant bacterial phyla (c) and genera (d, f) in the intestine of *T. ovatus* under different treatments. LDA score of LEfSe (e). Data are means ± SEM from three treatments (*n* = 3). Each parameter bar not sharing a common letter indicates a significant difference (*P* < 0.05).

As can be seen from Table 8, observed species and Shannon index in the D2 and D3 groups were higher than those in the D1 group. However, both Chao1 and Ace indexes showed no significant difference between the D2 and D1 groups (*P* > 0.05), while these indexes in the D3 group were significantly higher than those in the D1 group (*P* < 0.05). All the alpha diversity indices showed no significant difference between the D2 and D3 groups (*P* > 0.05). Interestingly, PCoA plots based on weighted UniFrac metrics (Figure 6(a)) and unweighted UniFrac metrics (Figure 6(b)) were further performed to confirm that the intestinal bacteria in the D1-D3 groups were clearly separated. Fish of the D2 group had the highest abundance of Firmicutes and the lowest abundance of Proteobacteria and Spirochaetota in the gut among the three dietary groups at the phylum level (Figure 6(c)), as well as had the highest proportion of *Bacillus* (Firmicutes) and the lowest proportions of *Photobacterium* (Proteobacteria) and *Brevinema* (Spirochaetota) at the corresponding genus level (Figure 6(d)). Furthermore, the proportions of *Mycoplasma* in the D2 group were significantly lower than that in the D3 group (*P* < 0.05, Figure 6(f)). Additionally, in the LEfSe LDA, *Photobacterium* was enriched in the D1 group, *Bacillus virus SPbeta* was enriched in the D2 group, and *Mycoplasma* and *Brevinema* were enriched in the D3 group (Figure 6(e)).

### 3.8. Association between the Intestinal Microbiota and Hepatic Metabolites

**Figure 7:**
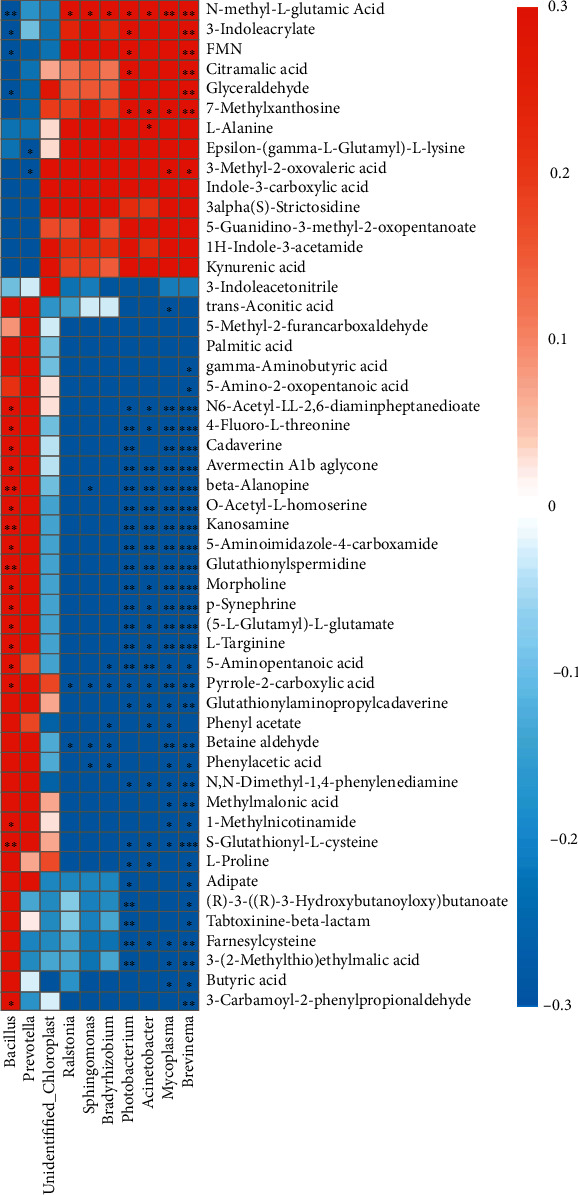
Significant correlation between intestinal bacteria at the genus level and hepatic DMs. The correlation coefficient is represented by different colours (red: positive correlation; blue: negative correlation). ^∗^Represents significantly negative or positive correlations (^∗^*P* < 0.05;  ^∗∗^*P* < 0.01;  ^∗∗∗^*P* < 0.001).

Pearson correlation analysis was conducted to reveal the relationships between intestinal microbial and hepatic metabolite (Figure 7). The intestinal probiotics (*Bacillus*) were significantly positively correlated with the changes in pyrrole-2-carboxylic acid, l-targinine, and p-synephrine (*P* < 0.05). On the contrary, the pathogenic bacteria (*Photobacterium*, *Brevinema*, and *Mycoplasma*) were significantly negatively correlated with changes in pyrrole-2-carboxylic acid, l-targinine, and p-synephrine. Moreover, *Brevinema* and *Mycoplasma* were significantly negatively correlated with changes in butyric acid.

## 4. Discussion

### 4.1. Better Fatty Acid Composition of BO1 Is the Main Reason for *T. ovatus* Fed Diet D2 Displaying Better Growth Performance

Our previous study has demonstrated that the proper dietary n-3 LC-PUFA level is 0.64%-2.10%, and the DHA/EPA ratio is about 1.4 for maintaining the normal growth, development, and survival of *T. ovatus* [8, 10]. In this study, the n-3 LC-PUFA content of diet D2 and D3 was 1.01% and 1.08%, respectively, which were in line with the demand level. However, the growth performance of fish fed D2 was better than that fed D3 and was comparative to that fed D1. Since the only difference between D2 and D3 is that their lipid sources and corresponding fatty acid compositions are different, the better growth performance of D2 than D3 indicates the importance of dietary fatty acid composition and precise nutrition. It is well known that the levels of dietary LA and n-6 PUFA and DHA/EPA ratio compromise the healthy growth of fish [10, 21–24]. In this study, the LA and n-6 PUFA levels (of total fatty acids) and the DHA/EPA ratio of diet D2 (14.47%, 15.28%, and 1.08) are close to those of D1 (9.67%, 11.17%, and 0.92). However, the corresponding values of D3 (24.72%, 25.56%, and 1.74) are obviously higher than those of D2. Besides, the DHA/EPA ratio of D3 not only was higher than that of D1 (0.92) and D2 (1.08) but also exceeded the proper ratio (about 1.4) or the limitation (1.69) for normal growth of *T. ovatus*; the growth was obviously inhibited when the dietary DHA/EPA ratio reached 1.69 [10]. Moreover, it has been reported that low SFA and high n-6 PUFA levels in diets would cause deficient energy supply and increasing lipid accumulation in the liver, which may consequently impair the growth performance of juvenile tiger puffer (*Takifugu rubripes*) [22]. In this study, the SFA proportion of D3 (31.16%) was lower than that of D1 (37.24%) and D2 (41.60). All the above data suggested that dietary LA, SFA, and n-6 PUFA levels and DHA/EPA ratio were important to *T. ovatus*, and their proper values are the important reasons for the better growth performance of fish fed D2.

Fish weight gain is mainly attributed to protein deposition [25]. In the present study, fish of the D2 group (high oleic acid and MUFA levels) had higher protein content in whole-body and muscle and lower lipid content than those of the D1 and D3 groups (high n-3 and n-6 PUFA levels, respectively), which is partly in agreement with previous researches where the replacement of dietary FO with vegetable oils like canola oil, containing a high level of oleic acid, may promote protein retention resulting in protein-sparing effects, through better fatty acid oxidation to provide energy for fish [26], while high levels of n-6 PUFA were observed to inhibit amino acid biosynthesis and reduce protein content in tiger puffer [22]. A high dietary n-3 LC-PUFA level could decrease the expression level of *pparα* and *cpt1a*, which contributes to inhibiting *β*-oxidation in the liver of juvenile black sea bream (*Acanthopagrus schlegelii*) as well [27]. Besides, diets rich in LA and n-6 PUFA were also found to increase lipid deposition in muscle of blunt snout bream (*Megalobrama amblycephala*) [28]. The above results suggested that a positive effect of BO1 on growth and protein deposition in *T. ovatus* may mainly owe to the better fatty acid compositions of BO1.

### 4.2. Possible Mechanisms of Dietary Fatty Acid Composition Affecting Growth Performance of *T. ovatus*

#### 4.2.1. *T. ovatus* Fed BO1 Diet Displayed Lower Oxidative Stress and Inflammatory Responses than Fish Fed BO2 Diet

It was reported that the antioxidation or oxidation state of fish is closely related to its health [29]. It is well known that MDA is a secondary oxidation product of PUFA, which has strong biotoxicity capable of damaging cell structure and function [30] and is frequently used as an indicator of lipid peroxidation to cell damage [31]. In the present study, the serum MDA content in fish fed D2 was lower than that in fish fed D3, which indicated that fish in the D2 group have the lowest oxidative stress. Meanwhile, fish fed D2 has lower GSSG content and SOD activity than those in fish fed D3. Previous studies demonstrated that the decreasing peroxidative damage by reactive oxygen species (ROS) results in decreasing activity of the antioxidant enzymes [32, 33]. Thus, the decrease of antioxidation enzyme activities also demonstrated that fish fed diet with BO1 as lipid had lower oxidative stress than those fed diet with BO2 as lipid in *T. ovatus*. The results may be due to the lower n-6 PUFA proportion in the liver of the D2 group than that of the D3 group in this study. Previous studies have showed that excess n-6 PUFA in diets or tissues can cause lipid peroxidation and oxidative stress [22]. Besides, results in this study have suggested that fatty acid composition in tissue of *T. ovatus* reflected dietary fatty acid profiles, which were consistent with the previous studies in other fish, such as silvery-black porgy (*Sparidentex hasta*) [26], gilthead sea bream (*Sparus aurata*) [34], and pearl gourami (*Trichogaster leeri*) [35]. Thus, the high level of n-6 PUFA in the diet could lead to the high deposition of these fatty acids in tissue, possibly resulting in stronger oxidative stress. In addition, studies have demonstrated that high levels of LA in diets could induce strong inflammation in fish, which leads to retarded growth [23, 24]. This viewpoint was further evidenced by the fact that fish fed D2 containing lower LA levels presented lower mRNA levels of proinflammatory factors, such as TNF-*α*, IL-1*β*, IL-8, IL-12, and IL-18, in the liver than that of fish fed D3 with higher LA levels in this study. The results suggested that *T. ovatus* fed BO1 diet displayed lower oxidative stress and inflammatory responses than fish fed BO2 diet.

#### 4.2.2. BO1 Diet Could Enhance Hepatic Immune-Related Metabolites and Improve the Immune Response of *T. ovatus*

Accumulating data indicate that there is a close relationship between metabolism and immune response [24, 36, 37]. In this study, hepatic metabolomic analysis indicated that dietary fatty acids affected the metabolic function of *T. ovatus*, especially amino acid metabolism. Among them, some immune-related DMs were identified in the liver. Some amino acids and their derivatives such as valine, gamma-aminobutyric acid (GABA), pyrrole-2-carboxylic acid, tyramine, and l-targinine, as well as phenols (p-synephrine) and short-chain fatty acid (butyric acid), were markedly increased in the D2 group compared with the D3 group (Table 7). It has been proved that the increase of valine, GABA, and pyrrole-2-carboxylic acid is related to the decreased levels of oxidative stress [38]. Furthermore, GABA can improve the antioxidant activity [39], and the GABAergic system (GABA, GABA receptors, glutamic acid decarboxylase, and GABA transporters) may be closely associated with the activation and function of immune cells [40]. Tyramine is a neuroactive chemical. Studies have shown that exogenous tyramine can enhance the immunity and resistance ability of *Litopenaeus vannamei* [41]. l-Targinine is a nitric oxide synthase inhibitor [42], which can prevent overproduction of NO causing cytotoxic effects [43]. p-Synephrine can suppress inflammatory responses by reducing reactive oxygen species and proinflammatory factor concentrations, such as TNF-*α* and IL-6 [44]. Butyric acid can improve the growth performance, nutrient utilization, intestinal health status, antioxidant activity, nonspecific immune responses, and the resistance of fish against the invading pathogens [45]. Therefore, the increase of the above metabolites in the liver of *T. ovatus* indicated that the BO1 diet may promote fish's growth by increasing the content of immune-related metabolites in the liver and improving its immune response.

#### 4.2.3. BO1 Diet Could Improve Intestinal Microbial Communities

The intestinal microbiota plays essential roles in host growth and immune and metabolic function [46]. In this study, the intestinal bacterial diversity of *T. ovatus* fed D2 and D3 showed no difference, but the bacterial communities in different dietary groups formed different clusters, indicating an obvious effect of lipid source, which was in line with our previous study in *T. ovatus* [12]. Fish of the D2 group had higher abundance of Firmicutes in gut and lower Proteobacteria proportion than that of the D3 group at the phylum level and also higher proportion of *Bacillus* and lower proportions of *Mycoplasma* and *Brevinema* and *Photobacterium* than that of the D3 group at the genus level in this study. It has been reported that Firmicutes, including vast beneficial bacteria, is essential for host to digest and absorb the nutrients [20, 47], and takes part in short-chain fatty acid synthesis [46] and improves lipid metabolism to facilitate energy harvest [48]. *Bacillus*, belonging to Firmicutes, is a typical probiotic and plays an important role in improving digestive enzyme activity, extracting nutrients consumed in feed, and enhancing host immunity [49–52]. Zhang et al. [53] also reported that the addition of *Bacillus subtilis* in diet can enhance the growth performance, immune responses, and disease resistance of juvenile *T. ovatus*. On the contrary, Proteobacteria is a marker for dysbiosis in gut microbiota (unstable microbial community) and a potential diagnostic criterion for disease, such as inflammation [54]. *Photobacterium*, *Brevinema*, and *Mycoplasma* belong to Proteobacteria, Spirochaetota, and Firmicutes, respectively, and are all regarded as pathogenic bacteria [55–58]. Thus, the increasing proportion of probiotics and the reducing proportion of pathogenic bacteria in gut of the D2 group indicated a better bacteria community composition of fish fed D2, which may also be another important factor of BO1 to display its excellent effects.

#### 4.2.4. Correlation between Intestinal Microbial Changes and Hepatic Metabolites

Intestinal microbial changes of host are usually associated with the changes in the host's metabolic profile [59]. In this study, the increased level of *Bacillus* (a typical probiotic) was positively associated with the changes in pyrrole-2-carboxylic acid, l-targinine, and p-synephrine; the decreased levels of *Mycoplasma*, *Brevinema*, and *Photobacterium* (pathogenic bacteria) were significantly negatively correlated with changes in pyrrole-2-carboxylic acid, l-targinine, and p-synephrine; the decreased levels of *Brevinema* was significantly negatively correlated with changes in GABA and butyric acid in the liver of *T. ovatus.* It has been demonstrated that all of the above metabolites correlated to immunity [38, 40, 43–45]. The correlation between intestinal microbial changes and these metabolites indicated that intestinal bacteria might be involved in the immune response of *T. ovatus*. Similarly, intestinal bacteria affected the immunity of host in *L. vannamei* [17]. The present results indicated that BO1 could enhance the immune-related metabolites of the liver by improving the intestinal microbial community, thus enhancing the immune response of *T. ovatus*.

In conclusion, the present study demonstrated that diet with BO1 as the lipid source can improve the growth performance of *T. ovatus*. Reducing oxidative stress and inflammatory responses, enhancing hepatic immune-related metabolites, and improving intestinal microbial communities may be the mechanism of BO1's excellent effect. Furthermore, intestinal bacteria might affect liver metabolism and participate in the immune response of *T. ovatus*. Since the only difference among D1-D3 is that their lipid sources and corresponding fatty acid compositions are different, and BO1 was developed according to the EFA demand characteristics of *T. ovatus*; we can thus conclude that the better performance of BO1 than that of BO2 indicates the importance of fatty acid precision nutrition.

## Figures and Tables

**Table 1:** Ingredients and proximate composition of the experimental diets.

Ingredients (g kg^−1^)	Diets		
	D1	D2	D3
Fish meal	120	120	120
Basic protein^1^	329	329	329
Compound protein^2^	180	180	180
Strong flour	170	170	170
Fish oil	80	—	—
Blend oil 1^3^	—	80	—
Blend oil 2^4^	—	—	80
Premix compound^5^	40	40	40
DL-Methionine	1.8	1.8	1.8
L-Lysine	3.8	3.8	3.8
Wheat bran	75.4	75.4	75.4
Proximate composition (% dry weight)			
Moisture	7.29	7.13	7.68
Crude protein	45.27	45.50	45.58
Crude lipid	12.87	13.15	12.84
Ash	9.20	8.98	8.90

Note: ^1^consisting of chicken meal, soy protein concentrate, and corn gluten meal; ^2^consisting of several terrestrial animal and plant proteins in different proportions as described in our previous study [14]. ^3^Consisting of fish, soybean, rapeseed, and Perilla oils in a certain proportion according to the *T. ovatus*' demand characteristics for essential fatty acids. The specific proportion is not showed due to the application of a patent. ^4^Consisting of 40% fish oil and 60% soybean oil. ^5^Including vitamin mixture and mineral compound, choline chloride, and monocalcium phosphate.

**Table 2:** Fatty acid composition of the experimental diets.

Main fatty acids (% total fatty acids)	Diets		
	D1	D2	D3
14:00	6.28	3.40	3.42
16:00	22.33	29.72	20.43
18:00	5.73	6.89	5.36
SFA	37.24	41.60	31.16
14:1	0.59	0.35	0.46
16:1	4.89	2.59	2.97
18:1	17.63	26.31	22.37
MUFA	26.29	30.21	27.91
18:2n-6 (LA)	9.67	14.47	24.72
20:4n-6 (ARA)	0.34	0.16	0.17
n-6 PUFA	11.17	15.28	25.56
18:3n-3 (ALA)	1.99	1.86	3.68
20:5n-3 (EPA)	8.81	3.70	3.05
22:6n-3 (DHA)	8.12	3.98	5.32
n-3 PUFA	20.19	10.06	12.57
n-3 LC-PUFA	16.93	7.67	8.38
DHA/EPA	0.92	1.08	1.74

Note: SFA: saturated fatty acids; MUFA: monounsaturated fatty acids; PUFA: polyunsaturated fatty acid; LC-PUFA: long-chain PUFA.

**Table 3:** Nucleotide sequences of the primers used to assay gene expressions by real-time PCR.

Target gene	Forward primers (5′-3′)	Reverse primers (5′-3′)	References
*il-1β*	GGACAGTCTGCTGGACGAAA	GTTGGTGAGCGTCCACCTTA	Genome sequences
*il-8*	GAGAAGCCTGGGAATGGAGC	CACTTTCTCAATGTGGCGGC	Genome sequences
*il-12*	ACCAGTGTCCAGCATTGGAG	GTGTCCGCTCTCGTCTACTG	Genome sequences
*il-18*	TGCAGCGGGGAGAATGAAAT	ACGCAAGACCAGTTGGTGAA	Genome sequences
*tnf-α*	TCGTGATCCCTCAACAAGGC	GTCTTTTGGCAGGCAGTTCG	Genome sequences
*β-Actin*	TACGAGCTGCCTGACGGACA	GGCTGTGATCTCCTTCTGC	[15]

Note: *il-1β*: interleukin-1*β*; *il-8*: interleukin-8; *il-12*: interleukin-12; *il-18*: interleukin-18; *tnf-α*: tumor necrosis factor *α*.

**Table 4:** Growth performance and feed utilization of *T. ovatus* fed different diets.

Items	Diets		
	D1	D2	D3
Initial weight	7.67 ± 0.17	7.67 ± 0.00	7.58 ± 0.09
Final weight	68.18 ± 2.51	71.55 ± 2.39	62.64 ± 2.16
WGR^1^	788.7 ± 13.92^ab^	833.29 ± 31.15^b^	726.49 ± 7.52^a^
SGR^2^	3.47 ± 0.02^ab^	3.54 ± 0.05^b^	3.35 ± 0.07^a^
FCR^3^	1.54 ± 0.06	1.46 ± 0.02	1.58 ± 0.05
SUR^4^	94.44 ± 2.22	100 ± 0.00	100 ± 0.00

Note: data represent means ± SEM from three treatments of fish (*n* = 3). In each row, data without sharing a common superscript letter indicates significant difference (*P* < 0.05). ^1^WGR: weight gain rate (%); ^2^SGR: specific growth rate (% day^−1^); ^3^FCR: feed conversion ratio; ^4^SUR: survival rate (%).

**Table 5:** Whole-body and muscle proximate composition of *T. ovatus* fed different diets.

Items	Diets		
	D1	D2	D3
Whole-body (%)			
Moisture	63.29 ± 0.56^a^	65.79 ± 0.39^b^	63.36 ± 0.54^a^
Protein	17.51 ± 0.14^a^	17.97 ± 0.13^b^	17.55 ± 0.09^a^
Lipid	15.60 ± 0.74^b^	12.88 ± 0.64^a^	15.63 ± 0.42^b^
Ash	3.90 ± 0.15	3.98 ± 0.10	3.81 ± 0.12
Muscle (%)			
Moisture	73.12 ± 0.27	73.85 ± 0.28	73.37 ± 0.42
Protein	19.24 ± 0.10^a^	19.97 ± 0.11^b^	18.92 ± 0.26^a^
Lipid	6.93 ± 0.18^b^	5.22 ± 0.22^a^	6.99 ± 0.48^b^
Ash	1.42 ± 0.03	1.28 ± 0.06	1.32 ± 0.04

Note: data represent means ± SEM from three treatments of fish (*n* = 3). In each row, data without sharing a common superscript letter indicates significant difference (*P* < 0.05).

**Table 6:** Fatty acid composition in liver of *T. ovatus* fed different diets.

Main fatty acids (% total fatty acids)	Diets		
	D1	D2	D3
16:00	30.47 ± 0.56^b^	29.37 ± 0.25^ab^	28.75 ± 0.36^a^
18:00	9.90 ± 0.76	10.02 ± 0.20	10.05 ± 0.29
SFA	43.44 ± 0.87	41.78 ± 0.44	41.53 ± 0.85
16:1	2.69 ± 0.13^b^	1.77 ± 0.05^a^	1.75 ± 0.06^a^
18:1	28.63 ± 0.94^a^	31.76 ± 0.13^b^	27.26 ± 1.00^a^
MUFA	36.89 ± 0.77^b^	38.31 ± 0.24^b^	34.01 ± 0.89^a^
18:2n-6 (LA)	4.08 ± 0.26^a^	6.36 ± 0.16^b^	9.58 ± 0.61^c^
20:2n-6	0.83 ± 0.08^a^	1.65 ± 0.07^b^	2.38 ± 0.12^c^
20:4n-6 (ARA)	0.33 ± 0.04^b^	0.17 ± 0.00^a^	0.15 ± 0.02^a^
n-6 PUFA	5.99 ± 0.38^a^	9.08 ± 0.23^b^	13.14 ± 0.79^c^
18:3n-3 (ALA)	2.48 ± 0.07^a^	3.33 ± 0.11^b^	3.36 ± 0.13^b^
20:3n-3	0.76 ± 0.08^a^	1.15 ± 0.03^b^	1.05 ± 0.02^b^
20:5n-3 (EPA)	0.71 ± 0.06^b^	0.18 ± 0.01^a^	0.14 ± 0.01^a^
22:6n-3 (DHA)	4.93 ± 0.31^b^	2.78 ± 0.10^a^	3.00 ± 0.29^a^
n-3 PUFA	10.20 ± 0.49^b^	7.92 ± 0.21^a^	7.98 ± 0.30^a^
n-3 LC-PUFA	5.64 ± 0.36^b^	2.95 ± 0.11^a^	3.13 ± 0.30^a^
DHA/EPA	7.08 ± 0.43^a^	15.72 ± 0.34^b^	22.49 ± 1.56^c^

Note: data represent means ± SEM from three treatments of fish (*n* = 3). In each row, data without sharing a common superscript letter indicates significant difference (*P* < 0.05). Abbreviations are consistent with Table 2.

**Table 7:** Significantly differential metabolites in liver of *T. ovatus* fed different diets.

Metabolite name	D2 vs. D3		D1 vs. D2		D1 vs. D3		Categories
	log2 (FC)	*P*	log2 (FC)	*P*	log2 (FC)	*P*	
2-Phenylacetamide	0.89	0.020	—	—	—	—	Amino acid
5-Amino-2-oxopentanoic acid	1.73	0.020	—	—	2.16	0.005	
(5-L-Glutamyl)-L-glutamate	22.16	0.003	—	—	22.26	0.003	
Asymmetric dimethylarginine	0.71	0.031	—	—	—	—	
Cadaverine	19.32	0.003	—	—	19.71	0.003	
Epsilon-(gamma-L-glutamyl)-L-lysine	-2.41	0.013	—	—	—	—	
Gamma-aminobutyric acid	0.65	0.013	-0.48	0.005	—	—	
Glutathionylaminopropylcadaverine	3.55	0.031	—	—	—	—	
Glutathionylspermidine	20.05	0.003	—	—	—	—	
Kynurenic acid	-1.78	0.020	—	—	—	—	
L-Proline	1.97	0.005	-1.63	0.005			
L-Targinine	20.54	0.003	—	—	21.36	0.003	
L-Valine	0.42	0.045	—	—	—	—	
Methylmalonic acid	2.43	0.020	-2.55	0.031	—	—	
N6-Acetyl-LL-2,6-diaminoheptanedioate	3.07	0.005	—	—	2.21	0.005	
O-Acetyl-L-homoserine	19.53	0.003	—	—	20.16	0.003	
Phenylacetic acid	1.70	0.020	-1.04	0.045	—	—	
Pyrrole-2-carboxylic acid	5.14	0.005	-2.65	0.005	2.49	0.013	
S-Glutathionyl-L-cysteine	2.85	0.005	-1.49	0.005	1.36	0.013	
Tyramine	0.29	0.020	—	—	—	—	

(2R)-2-Hydroxy-3-(phosphonatooxy)propanoate	-0.81	0.045	—	—	—	—	Carbohydrate
D-Lyxose	-1.49	0.031	—	—	—	—	
Glyceraldehyde	-3.17	0.005	1.15	0.045	-2.02	0.005	
Glyceric acid	-1.31	0.045	—	—	—	—	
Maleic acid	-1.40	0.045	—	—	—	—	
Maltotetraose	1.27	0.031	—	—	—	—	
N-Acetyl-D-glucosamine	-0.68	0.013	—	—	-0.84	0.005	
N-Acetylmannosamine	-0.44	0.031	—	—	-0.51	0.013	
Trans-aconitic acid	2.43	0.045	-2.42	0.031	—	—	

1-Methylnicotinamide	1.60	0.008	-1.48	0.031	—	—	Cofactors and vitamins
FMN	-1.50	0.005	—	—	-1.59	0.005	
Pyridoxamine	1.36	0.020	—	—	1.43	0.005	
Pyridoxal	-0.80	0.045	—	—	-1.24	0.020	

Hydrogen phosphate	-0.41	0.020	—	—	-0.34	0.005	Energy

2-Hydroxybutyric acid	0.98	0.005	—	—	0.85	0.031	Lipid
3-Methyl-3-hydroxypentanedioate	1.18	0.045	—	—	—	—	
6-Hydroxyhexanoic acid	1.06	0.005	—	—	—	—	
Avermectin A1b aglycone	23.03	0.003	—	—	—	—	
Butyric acid	1.93	0.045	-2.16	0.031	—	—	
Citramalic acid	-2.07	0.005	2.19	0.008	—	—	
Glycerol	-0.90	0.008	—	—	-0.61	0.020	
Kanosamine	21.66	0.003	—	—	21.23	0.003	
Phosphorylcholine	-0.78	0.005	—	—	-0.98	0.005	

Xanthylic acid	-0.90	0.005	—	—	-1.29	0.008	Nucleotide

p-Synephrine	19.73	0.003	—	—	19.96	0.003	Phenols

4-O-Methylnorbelladine	1.49	0.031	—	—	—	—	Secondary metabolite

7-Methylxanthosine	-1.97	0.005	—	—	-1.92	0.005	Xenobiotics
Adipate	2.42	0.005	-2.25	0.005	—	—	
Morpholine	18.46	0.003	—	—	—	—	
Theobromine	-1.37	0.031	—	—	—	—	

Thiabendazole	-0.69	0.005	0.44	0.008			NI
N,N-Dimethyl-1,4-phenylenediamine	1.66	0.020	—	—	1.49	0.005	
Octanal	0.40	0.020	—	—	—	—	
3-Hydroxypicolinic acid	0.92	0.008	—	—	—	—	
Phenyl acetate	1.88	0.045	—	—	—	—	
2-Methylserine	1.30	0.020	—	—	—	—	
(S)-Piperidine-2-carboxamide	0.87	0.031	—	—	1.09	0.013	
4,5,6,7-Tetrahydroisoxazolo(5,4-c)pyridin-3-ol	1.07	0.013	—	—	1.17	0.013	
4-Fluoro-L-threonine	20.25	0.003	—	—	—	—	
7-Methylguanine	-0.93	0.045	—	—	-0.98	0.045	

Note: the differential metabolites (DMs) were identified according to *P* < 0.05 and the first principal component of variable importance in projection (VIP) values (VIP > 1.54). FC: fold change; NI: no identification.

**Table 8:** Alpha diversity indices of intestinal microbial of *T. ovatus* fed different diets.

Items	Diets		
	D1	D2	D3
Observed species	319 ± 6.34^a^	394.25 ± 28.95^b^	438.75 ± 15.90^b^
Shannon	3.62 ± 0.16^a^	4.36 ± 0.08^b^	4.43 ± 0.11^b^
Chao1	471.84 ± 22.57^a^	592.71 ± 74.01^ab^	678.91 ± 32.84^b^
Ace	382.2 ± 12.94^a^	404.32 ± 35.08^ab^	516.71 ± 21.66^b^
Coverage (%)	99.86 ± 0.03	99.87 ± 0.01	99.83 ± 0.01

Note: data represent means ± SEM from three treatments of fish (*n* = 3). In each row, data without sharing a common superscript letter indicates significant difference (*P* < 0.05). Alpha diversity indices, including observed species, Shannon, Chao1, Ace, and coverage, reflect community richness, community diversity, and community coverage.

## Data Availability

All data to support the findings of this study are included in this paper.
